# Comparative Characterization of Hot-Pressed Polyamide 11 and 12: Mechanical, Thermal and Durability Properties

**DOI:** 10.3390/polym13203553

**Published:** 2021-10-15

**Authors:** Mohsen Bahrami, Juana Abenojar, Miguel Angel Martínez

**Affiliations:** 1 Materials Science and Engineering and Chemical Engineering Department, University Carlos III de Madrid, 28911 Leganes, Spain; abenojar@ing.uc3m.es (J.A.); mamc@ing.uc3m.es (M.A.M.); 2 Mechanical Engineering Department, ICAI, Universidad Pontificia Comillas, 28015 Madrid, Spain

**Keywords:** polyamides, PA11, PA12, hot press, characterization, semi-crystalline, durability, water absorption

## Abstract

Chemically speaking, polyamide 11 (PA11) and polyamide 12 (PA12) have a similar backbone, differing only in one carbon. From an origin point of view, PA11 is considered a bioplastic polyamide composed from renewable resources, compared to oil-based PA12. Each of them has a number of advantages over the other, which makes their selection a challenging issue. Depending on the target application, diverse assessments and comparisons are needed to fulfill this mission. The current study addresses this research gap to characterize and compare PA11 and PA12 manufactured by the hot press technique in terms of their mechanical, thermal and durability properties for the first time, demonstrating their potential for future works as matrices in composite materials. In this regard, different characterization techniques are applied to the hot-pressed polymer sheets, including X-ray diffraction (XRD), differential scanning calorimetry (DSC), Fourier transform infrared spectroscopy (FTIR) and scanning electron microscopy (SEM). The mechanical performance of the PA11 and PA12 sheets is compared based on tensile tests and shore hardness measurement. The durability behavior of these two polyamides is evaluated in water and relative humidity conditions at different aging times. The experimental results show the ductile behavior of PA12 with respect to the quasi-brittle PA11. Both have a relatively small water and moisture gain: 1.5 wt% and 0.8 wt%, respectively. The higher crystallinity of PA12 (2.1 times more than PA11) with γ-phase is one of the leading parameters to achieve better mechanical and durability properties. The FTIR spectra displayed slight acid hydrolysis. Accordingly, absorbed water or moisture does not cause plasticization; thus, neither hardness nor dimension changes.

## 1. Introduction

Nylon is a term representing certain types of thermoplastic polymers belonging to the family known as polyamides. The term nylon, derived from a combination of New York and London, was suggested by Wallace Carothers, an early researcher of these materials at Du Pont Chemical Co. of America in 1928, who found that a pound of nylon could be converted to a length equal to the distance between New York (NY) and London (LON) [1,2]. A wide range of polyamides have been manufactured and marketed under various trade names. Generally, their names can be classified into two types, i.e., nylon XY, such as nylon 66, and nylon Z, such as nylon 6. X and Y refer to the number of carbon atoms in the diamine and diacid monomer, respectively. Moreover, Z in Z-type nylon refers to the number of carbon atoms in the monomer [1]. Polyamides can be divided into two general groups of aromatic and aliphatic. The latter polyamide is the paramount class of engineering thermoplastic polymers and shows the most impressive material characteristics compared to other polymers used in the industry [3]. These materials have been extensively investigated for decades in several industrial fields, including the automotive, textile, packaging, electric and electronics, sports and oil and gas industries, due to their unique combinations of properties, such as excellent durability [4] and mechanical strength [5], high-temperature and chemical resistance [6], ease of processing and high melting point [7]. Especially in recent years, the demand for PA products has increased significantly to replace specific metal structures in the fields of power tools, automotive and power train systems [8]. The chemical structure of polyamides mainly consists of amide groups that participate in hydrogen bonding resulting in reduced interchain mobility and accordingly causes high melting temperatures and high strength [9].

**Figure 1:**
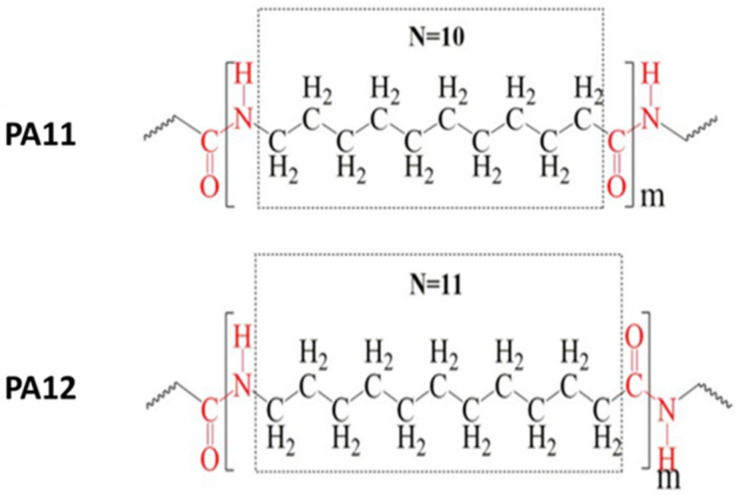
Chemical structure of PA11 and PA12 [12] (N: number of methylene groups between amide groups). Copyright 2020 Elsevier.

Along with PA6 and PA66, which constitute approximately 90% of the world’s overall polyamide utilization [10], PA11 and PA12 are other commercially notable members of the single-monomer nylon family. From a structural point of view, PA11 and 12, which are made up of amide groups separated by 10 or 11 CH_2_ groups (Figure 1), are constructed by a longer aliphatic chain length, a higher concentration of methylene groups and a lower amount of hydrogen bonds per unit of mass in comparison to other PAs [11].

PA12 is an oil-based thermoplastic polyamide with a semicrystalline structure [13]. It can be prepared from laurolactam monomer as well as the corresponding w-amino acid [14]. Depending on the crystallization conditions, it can demonstrate different crystalline forms. The main crystal form of γ, which has hexagonal packing with a strong diffraction peak, is obtained from melting at atmospheric pressure [15]. The α form with a monoclinic lattice gives two diffraction peaks with d-spacing of around 0.37 nm and 0.44 nm [16]. Koch and Jue [17] also reported two other crystalline forms of α’ and γ’. The performance of this polyamide is highly dependent on its crystal structure and morphology. PA12 possesses the outstanding mechanical properties of PA6 and 66, such as hardness, tensile strength, fatigue and impact resistance, a low coefficient of friction and resistance to aromatic hydrocarbons [14]. Moreover, it has the lowest melting point (which is still high enough for most practical purposes) among the polyamides and a lower moisture absorption capacity [18]. Due to its relatively long hydrocarbon chain, the density of PA12 is only 1.01 g/cm^3^. Based on the mentioned characteristics, PA12 and its copolymers are used in many industries, especially in sports goods [19] (e.g., soccer shoes [20]), aerospace [21,22,23], automotives [24] (e.g., fuel tubes [25] and under-the-hood [26]), food (e.g., packaging [27,28,29]), health (e.g., femoral stems [30], hip implants [31] and biomedical [32]) and the oil and gas (e.g., oil and gas pipelines [33,34] and crude oil transport [35]) industry.

On the other hand, PA11 is a bio-based polyamide synthesized from renewable resources such as castor oil that, compared to oil-based PA12, has a lower carbon footprint [6,36]. The progressive demand for this polyamide is not only due to its biocompatibility but also because of its high impact strength, high fatigue resistance [37], low moisture absorption [38], close-molding tolerance and excellent chemical and aging resistance [39]. PA11 is a semicrystalline polyamide with six different reported crystalline phases, including α, α’, δ, δ’, γ and β [40,41]. Among them, α, α’ have a triclinic structure and δ, δ’, γ have a pseudohexagonal one. The mechanical properties of this polyamide are highly affected by these phases and the degree of crystallinity [42]. Low moisture absorption is one of the critical features of PA11, which is caused by increasing the ratio of methylene to amide groups [18]. Moreover, due to the relatively low processing temperature of PA11 (approximately 185 °C), this polymer is widely used as a matrix in natural fiber-reinforced composites because of the low thermal stability of natural fibers [43,44,45,46,47].

These polyamides have been processed by different techniques. For example, PA12 and PA11 are the most extensively utilized polymers with some categories of powder bed fusion (PBF) techniques. [48]. Lanzl et al. [49] employed selective laser sintering (SLS, one of the PBF sub-techniques) to manufacture and characterize copper-filled PA12 in terms of thermal conductivity. One of the advantages of this processing technique is the ability to produce parts with high geometric complexity. Pandelidi et al. [50] characterized PA11 powders processed by the multi-jet fusing technique (MJF, another category of PBF techniques) and investigated the effects of PA11 refresh ratios on its mechanical performance. These two polyamides are favorable in these techniques due to the broad temperature range between their melting and crystallization temperature during heating and cooling, respectively. Moreover, PA11 and PA12 have excellent printability in 3D printing techniques. In the study of Rahim et al. [3], the mechanical properties of PA12 printed parts were improved by employing the fused deposition modeling (FDM) technique and incorporating bioceramic fillers. On the other hand, there is a common polymer-processing technology of injection molding. Jariyavidyanont et al. evaluated the skin/core structure of injection-molded PA11. They found that the gradient of the semicrystalline structure in the skin and core of the injected polymer did not lead to shear-induced crystallization effects [51]. In another study, Meyer et al. [52] examined the effect of melting temperature, as the critical process parameter of the injection molding technique, to produce plates of PA12.

The hot press technique is still a popular processing method to produce non-complex-shaped polymers. In comparison to injection molding, it can provide a higher molding pressure and longer annealing time, which leads to superior properties in some aspects [53]. Moreover, the shear-induced issue during injection molding, which causes deformation or molecule orientation, does not occur in the hot press method. In addition, this technique is still more feasible and cost-efficient with respect to fusion-based techniques. As far as we are aware, many authors have studied the properties of sintered, molded or lasered PA11 and PA12. However, there is still a lack of information regarding comparing these two commercial polyamides simultaneously in terms of their mechanical, thermal and durability properties when manufactured using the hot press technique. To compensate for this research gap, the present study investigated the tensile, hardness, thermal and durability properties of hot-pressed PA11 and PA12 simultaneously for the first time. It is worth mentioning that this research study is the initial step toward future works to employ these two thermoplastics as a matrix in hybrid composites with carbon and natural fibers.

## 2. Materials and Methods

### 2.1. Materials and Sample Preparation

**Figure 2:**
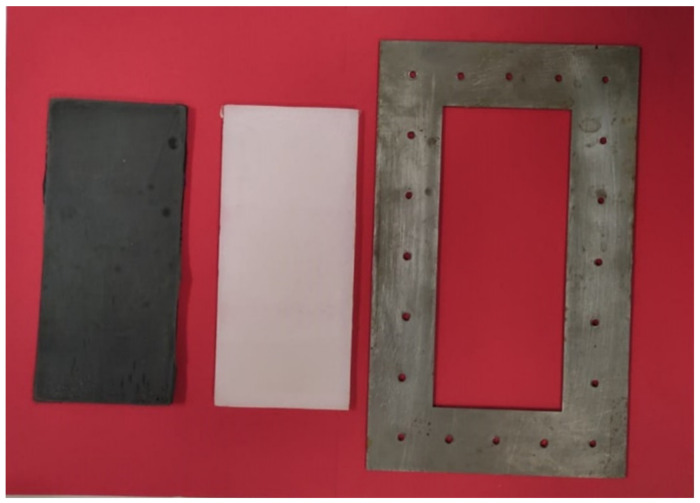
Hot-pressed PAs.

**Figure 3:**
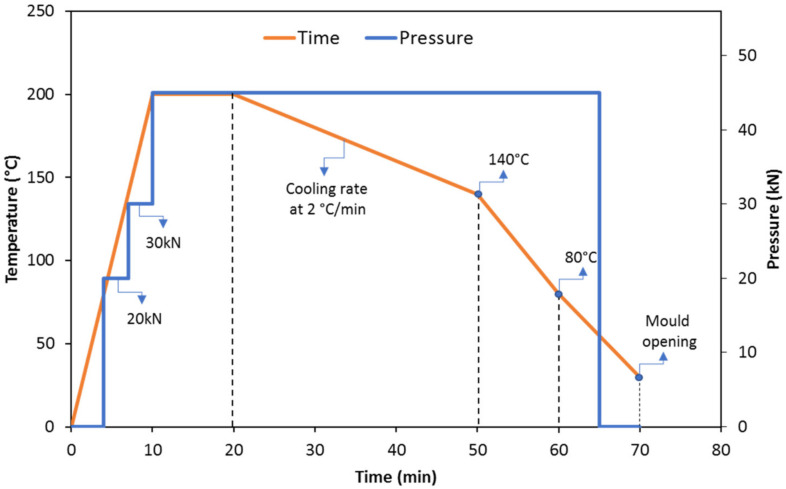
Hot press cycle for manufacturing PA samples.

Commercial thermoplastic polyamide pellets (PA11 and PA12) were provided by Arkema (Madrid, Spain). PA sheets and bulks were prepared by a hot plate press machine (Fontune Presses TPB374, Barendrecht, The Netherlands). PA pellets were filled inside the steel mold; the mold was then sandwiched between two aluminum sheets of 2 mm and two steel plates of 3 mm thickness to produce bulk and sheets of polyamides with 4 mm and 1 mm thickness, respectively (Figure 2). PA pellets were melted in the hot press with a maximum temperature and pressure of 200 °C and 45 kN. The molding cycle is shown in Figure 3. Gradual pressure steps were employed to obtain better densification and remove the trapped air between pellets. The temperatures of 200 °C (during the heating) and 140 °C (during the cooling) were selected to fulfill the melting point and end of the crystallization peak according to the DSC results.

### 2.2. Mechanical Test

The tensile test was performed using a Universal Hydraulic Tensile Test Machine (Microtest EM2/FR, Madrid, Spain), according to the ASTM D638 standard. Five specific samples of each polyamide were cut to be tested according to the standard specimen dimensions. Tests were carried out with a load cell of 10 kN under a 0.5 mm/min crosshead speed. The anomalous results of these five specimens were checked by the statistical method of Grubbs.

### 2.3. X-ray Diffraction

The XRD experiment was conducted to study the crystal details of both powder and sheet PAs. The X-ray diffraction patterns were acquired using Philips X’Pert diffractometer provided with a PW3011/10 detector (Amsterdam, Netherlands) at a voltage of 40 kV and a current of 40 mA. The monochromatic radiation CuKα (λ = 1.54 Å, 40 kV and 40 mA) was performed at room temperature in the 10° < 2θ < 30° interval, with a stepping angle of 0.02° and point acquisition time of 1 s.

### 2.4. Differential Scanning Calorimetric (DSC) Technique

DSC analysis (DSC 822e, Mettler Toledo GmbH, Greifensee, Switzerland) was performed on the three polymer sheets of each polyamide to examine the thermal properties of the specimens. The DSC was performed with a heating rate of 20 °C/min in a temperature range of −20 °C to 200 °C. Aluminum crucibles of 40 µL were used and filled with 8–10 mg of polyamide. Moreover, nitrogen as a purge gas was fed at a rate of 50 mL/min. The degree of crystallinity was obtained with Equation (1) [54]:

(1) $$X_{C}=\frac{\Delta H_{m}}{\Delta H_{m}{}^{o}}\times 100\%.\;$$

where X_c_ is the degree of crystallinity, ΔH_m_ is the melting enthalpy of the polymer and ΔH_m_^o^ is the melting enthalpy of the 100% crystalline PAs (ΔH_m_^o^, _PA11_: 244 J/g [55], ΔH_m_^o^, _PA12_: 95 J/g [56]).

### 2.5. Fourier Transform Infrared Spectroscopy (FTIR)

The FTIR spectra of the PA sheets were captured using an infrared spectrometer machine (Brucker Optic GmbH, Madrid, Spain). The produced spectra at approximately 5–10 μm depth were recorded with a Bruker Tensor 27 spectrometer, which used a diamond prism with a resolution of 4 cm^−1^, 32 scans and an incident radiation angle of 45°. The attenuated total multiple reflection technique (ATR) was used to analyze the surface chemical modifications (FTIR-ATR). Three spectra were recorded for each polymer to ensure homogenous results.

### 2.6. Durability Test

The water or moisture absorption is facilitated in polyamides due to the presence of polar amide groups, by forming hydrogen bonds with water molecules [57]. Water and moisture can affect the polymer characteristics in terms of mechanical, chemical and physical properties as well as dimensional stability [58]. Absorbed water or moisture reduces entanglement between polymer chains, leading to increased chain mobility. This behavior, called the plasticizing effect, even reduces the potential sites for inter-polyamide chain hydrogen bonding [59]. Accordingly, the strength and stiffness would be decreased [60]. In this study, the water and moisture absorption behavior of polymers in water and relative humidity were evaluated. In this regard, the bulk polyamide specimens according to the standard (ASTM D570-98) were cut to dimensions of 20 × 20 × 4 mm^3^ and were placed into two different containers. The PAs were immersed in distilled water at room temperature in the first container for the water absorption measurement. The second container was filled with distilled water and maintained at a constant temperature of 23 °C and relative humidity of 50%. Then, specimens were placed on a grid to prevent contact with water. For both tests, the specimens were removed from each container after aging times of 2 h, 5 h, 24 h, 168 h, 360 h, 744 h, 1512 h and 1656 h; afterward, excess water from the surface of the samples was dried gently with tissue paper and weighed immediately. After each removal, water and moisture absorption was evaluated by the relative uptake of weight, Mt, according to Equation (2):

(2) $$M_{t}=\frac{W_{t}-W_{0}}{W_{0}}\times 100$$

where W_0_ is the weight of the initial specimen in dry conditions and W_t_ is the weight of the wet specimen at each aging time. Three specimens were measured for each aging time. Furthermore, during the durability test, the DSC and FTIR analyses were carried out on the specimens with the aging times of 2 h, 24 h, 360 h, 744 h and 1656 h to evaluate the behavior of the polymers after water and moisture uptake.

### 2.7. Hardness Measurement

The hardness of the PA specimens before and after the durability test was determined using the Shore-D durometer (Bareiss Prüfgerätebau Gmb, Oberdischingen, Germany) as per ASTM D-2240. The measurements were carried out on 9 points of the specimens in accordance with the standard, and the accuracy of results was checked with the Grubbs method, similarly to the tensile test.

## 3. Results and Discussion

### 3.1. Tensile Strength

**Figure 4:**
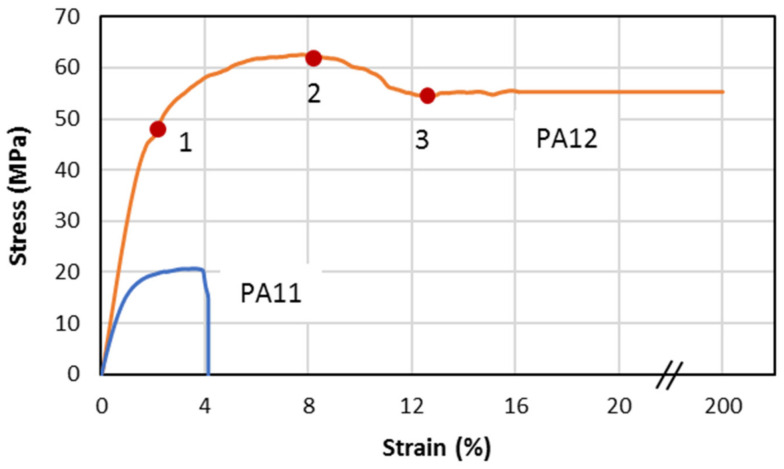
Stress–strain curves of PA11 and PA12 upon tensile test.

Figure 4 compares the stress–strain response of PA11 and PA12 under the tensile test. The tensile properties of PAs are also summarized in Table 1. PA11 showed quasi-brittle behavior; after the elastic deformation, the specimens underwent yielding followed by a plastic deformation region.

**Figure 5:**
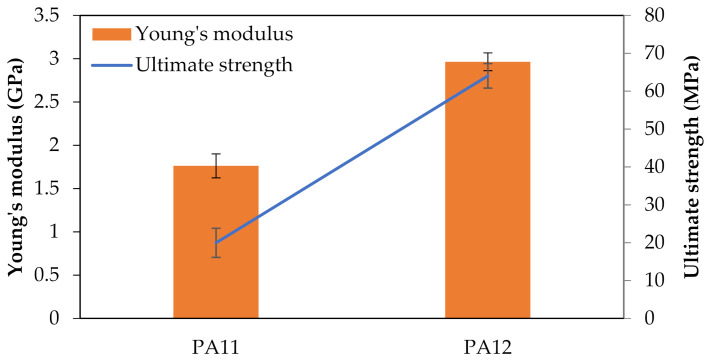
Comparison of tensile properties between PA11 and PA12.

**Figure 6:**
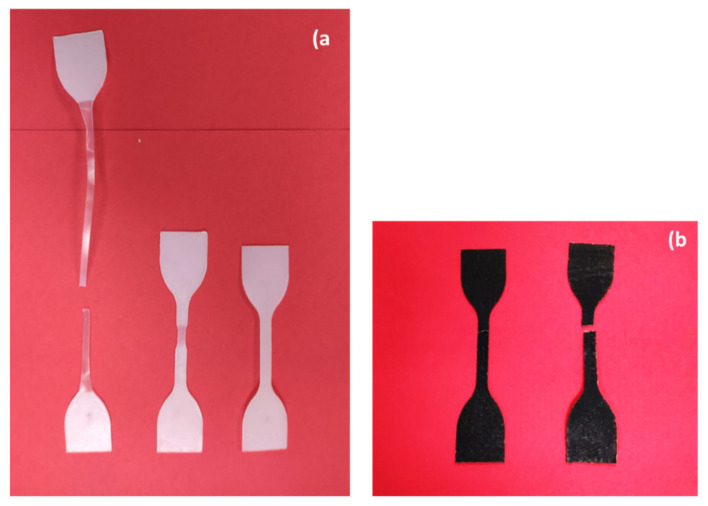
Comparison of tensile specimens: (**a**) PA12, (**b**) PA11.

On the other hand, PA12 demonstrated highly ductile behavior. Samples were stretched up to 200% before breaking because of the high resistance to deformation. They had higher tensile strength, Young’s modulus as well as elongation in comparison to PA11 (Figure 5). Points 1 and 2 are the first and second yield stress, which correspond to the plasticization and breakage of crystals, respectively [61]. Moreover, point 3 is the beginning of necking, and the whitening phenomenon is significant at this point for thermoplastics [62]. The mentioned behavior of specimens under tensile load are compared in Figure 6.

The difference in the tensile properties of these two PAs can be explained in terms of the degree of crystallinity. The tensile strength and elastic modulus are directly related to the stiffer part of the structure, i.e., the crystal phase. Accordingly, the higher degree of crystallinity leads to superior tensile strength and elastic modulus. As will be discussed in Section 3.3, the DSC results showed a higher degree of crystallinity in PA12, which validates the tensile test results.

SEM observations were performed on cross-sections of the fractured polymers after tensile loading. To better explain the SEM images, understanding the microstructure of PA11 and PA12 and the fraction mechanisms is essential. These polymers are semicrystalline, comprising crystalline and amorphous phases; thus, their microstructure is highly heterogenous. The crystalline lamella is connected to the amorphous region by non-entangled chains or taut tiemolecules [63,64]. Due to the significantly lower Young’s modulus of amorphous regions compared to crystal lamella, the first deformation usually occurs in the lamellar amorphous phase in the form of lamella separation, interlamellar shear and rotation of lamellar stacks. This usually happens at small strains before yielding [65]. Interlamellar separation causes strong hydrostatic tension within the constrained amorphous network, which leads to mixed fracture behavior. On one hand, this local stress can be relieved through cavitation within the amorphous network around the yielding point. On the other hand, simultaneously, the highly extended tie chains transfer concentrated forces to the crystal lamella. The latter results in slipping on the preferred lattice planes, lamella fragmentation, chain unfolding and/or crystal breakup [64,66].

**Figure 7:**
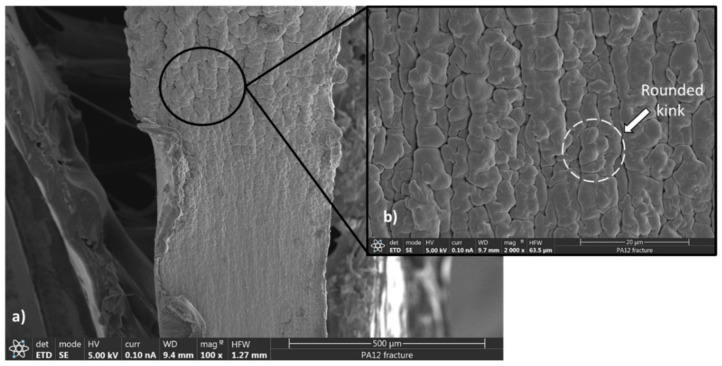
SEM micrographs of the PA12 fracture surface: (**a**) magnification of 100×, (**b**) magnification of 2000×.

**Figure 8:**
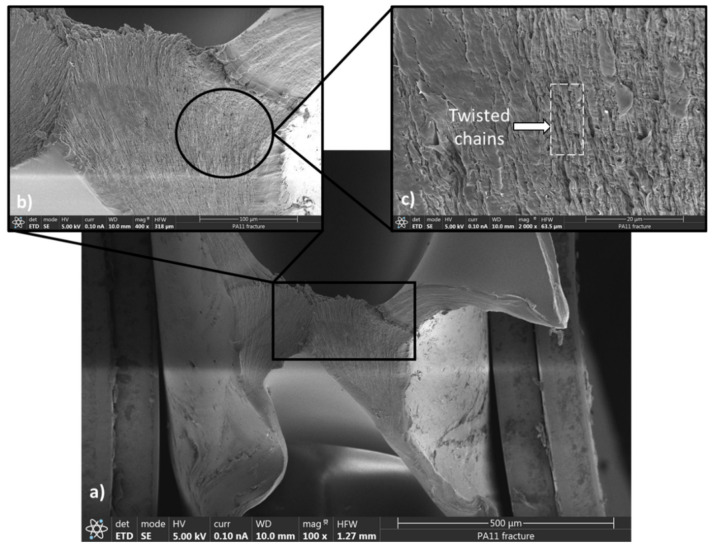
SEM micrographs of the PA11 fracture surface: (**a**) magnification of 100×, (**b**) magnification of 400×, (**c**) magnification of 2000×.

As illustrated in Figure 7a, the fragmentation appears within the waviness fracture surface of PA12 in the form of folds or rounded kinks of lamella. The large kinks (Figure 7b) are usually formed by significant plastic strain, which is typical for a ductile fracture. This fracture type was also reported once both slip in the crystals and deformation of the amorphous regions occurred simultaneously [67]. On the contrary, Figure 8a exhibits a smooth surface for the quasi-brittle fracture of PA11. According to Figure 8b,c, the deformation occurred in the form of chain tilting and twisting by slipping and orienting along the force direction under the tension.

Table 2 compares the tensile properties of the current study with previous works. PA12 exhibited improved tensile properties with respect to other studies. A higher degree of crystallinity would be an essential parameter for this achievement. Furthermore, the higher impact pressure of the hot press technique regarding injection molding and laser sintering resulted in a more compact structure with lower porosity and better tensile properties. As for PA11, although it had a higher elastic modulus, its ultimate strength was lower than in previous works. This can be explained by the more brittle behavior of the produced polymer in this study, besides the lower degree of crystallinity compared to the other works. The low elongation at break is evidence for this hypothesis. Moreover, this variance may be attributed to differences in the type of feedstock of PA11 and also the manufacturing methods. A better comparison might be obtained by considering the porosity percentage of each study, which is beyond the scope of the current study.

### 3.2. X-ray Diffraction

**Figure 9:**
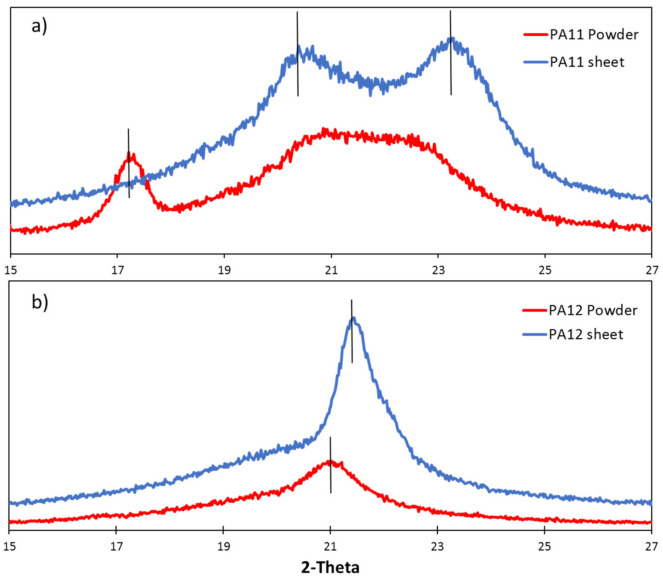
XRD patterns for PAs in the form of powder and sheets: (**a**) PA11, (**b**) PA12.

It is well-documented that the PAs investigated in this research have two different kinds of crystal forms: α crystals and γ crystals. PA12 has a monoclinic structure as α crystals, while PA11 has a triclinic structure as α crystals. Additionally, all of the PAs have a pseudohexagonal crystal structure as γ crystals. PA11 has a predominant amount of α crystals, while PA12 tends to contain γ crystals instead of α crystals [77]. Figure 9 exhibits the XRD patterns of PAs in the form of powder and sheets. The principal differences between the powder and sheet XRD results arise from the fact that powders are generally analyzed by Theta/2Theta geometry. In contrast, sheets typically are analyzed in grazing incidence geometry. Moreover, measurements on powders will always display a random orientation of the grains, whereas sheet crystals show more pronounced peaks for one lattice plane due to the preferred orientation, and all the others have substantially lowered or even absent peak intensities. In this regard, the sheet patterns are considered in this study.

As shown in Figure 9a, the XRD pattern of the PA11 sheet has an amorphous halo with two crystalline reflection peaks corresponding to the α phase at 2θ = 20.5° and 23.7° (Table 3), respectively, associated with the (100) and (010/110) planes with a “d spacing” of 0.432 nm and 0.375 nm, indicating the presence of the triclinic α-form of PA11 [78,79,80]. In the literature, it is reported that these two reflection peaks merge into a single reflection peak of pseudohexagonal γ-phase upon increasing the temperature [81,82]. Similarly, PA12 is a semicrystalline polymer, and therefore the PA12 sheet exhibits a crystalline peak at 21.45° indicating the γ-crystal form (100) corresponding to d spacings of 0.413 nm [83].

**Figure 10:**
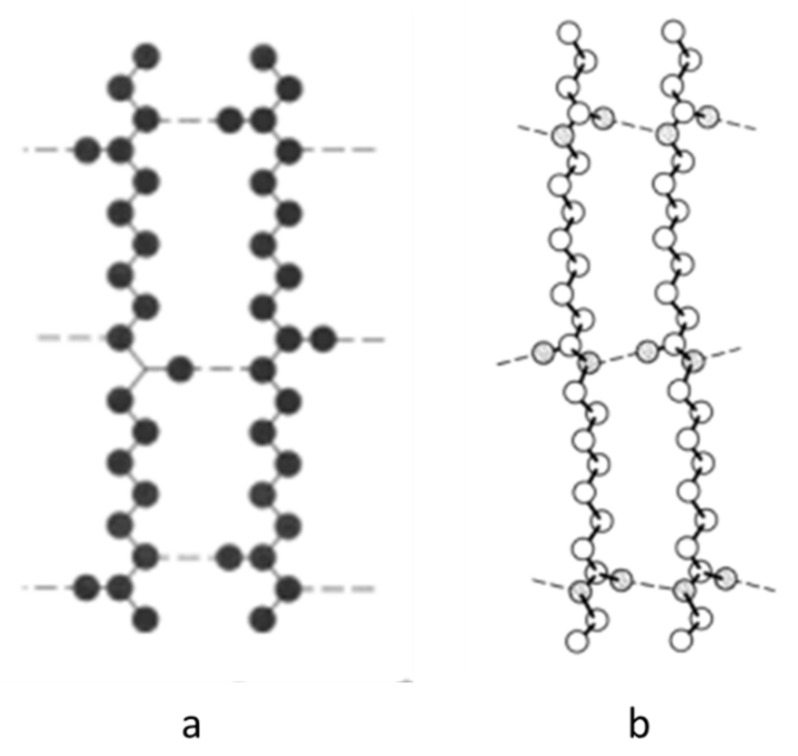
Chains in the (**a**) fully extended and (**b**) twisted configuration in PAs. Adapted from [84]. Copyright 1973 John Wiley and Sons.

In linear aliphatic polyamides, it is a controlling factor for crystalline phases that all the hydrogen bonds are completely satisfied [84]. To this aim, in PA11 and PA12, the chains would be adept in two different configurations (Figure 10) and can orient in a parallel or antiparallel way in the α and γ phase depending on the number of CH_2_ groups in the structure. Within the α phase of PA11, the chains appear in a fully extended configuration, and hydrogen bonds form a large molecular sheet by occurring between amides in the same plane as the CH_2_ zig-zags. Within the γ phase of PA12, the chains appear in a slightly twisted configuration, and hydrogen bonds occur between antiparallel chains [3,78,84]. Regarding the phase stability, the α and γ phase have close internal energy, reported from 0.045 to 1.30 kcal/mol as energy differences [85,86,87,88]. In computational studies, it was shown that for polyamides with less than six methylene units (*n* < 6), the α phase is more stable, and for those with more than six (*n* > 6), the γ phase is more stable [85,88]. Although this agrees with the results of the last part that PA12 with the γ phase is more stable and has higher tensile properties, most of the computational studies focus on defect-free crystals. Thus, due to the inevitable presence of defects and also the slight differences in phases’ internal energy, a direct relation between mechanical properties and the type of crystal phase is unreliable.

### 3.3. DSC Analyses

**Figure 11:**
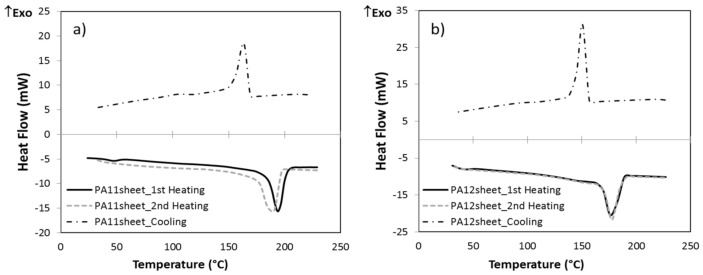
DSC thermograms of PA sheets (heating rate: 20 °C/min): (**a**) PA11, (**b**) PA12.

Thermal properties were measured by DSC for sheets of PA11 and PA12. The thermographs obtained during the first and second heating are depicted in Figure 11. On the right-hand side of the heating curves, the sharp endothermic peaks present a melting point, and on the left-hand side, the lower-temperature zone shows the glass transition. Moreover, the exothermic peaks in cooling curves are assigned to the recrystallization process. Melting temperature (T_m_), recrystallization temperature (T_c_), melting enthalpy (ΔH) and degree of crystallinity (X_c_), which were measured based on Equation (1), are shown in Table 4. The degree of crystallinity of PA12 is around 2.1 times more than PA11, which is one of the reasons for the higher tensile properties and lower water absorption of PA12. Moreover, PA12 has a greater recrystallization enthalpy than melting enthalpy, which produces a higher melting peak and a slight increase in T_g_ within the second heating. For PA11, the opposite takes place.

**Figure 12:**
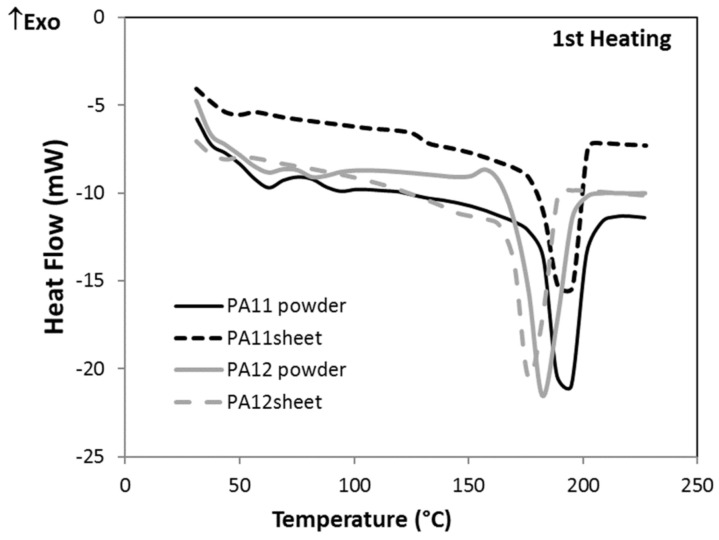
DSC thermograms of PA powders and sheets in the first heating cycle.

Furthermore, Figure 12 compares the first heating cycle of PA powders with their sheets. It was observed that after hot pressing the PA11 powder, T_g_ decreased and X_c_ increased. The thermal history of powder is erased by heating in the hot press; then, the enthalpy of relaxation (known as aging) disappears, and any water adsorbed on the surface is lost. Since the cooling in the hot press is slow, there is more time for crystallization; thus, the crystallinity increases and T_g_ decreases. For PA12, in addition to the mentioned explanation, recrystallization must occur in the hot press before melting due to the heat effect. However, during DSC, this process is not known. Thus, the T_g_ decreased, and the crystallinity was the same as that of the powder. The powders’ thermal properties as determined by DSC are presented in Tables S1 and S2 of the Supplementary Materials.

### 3.4. FTIR-ATR Analyses

**Figure 13:**
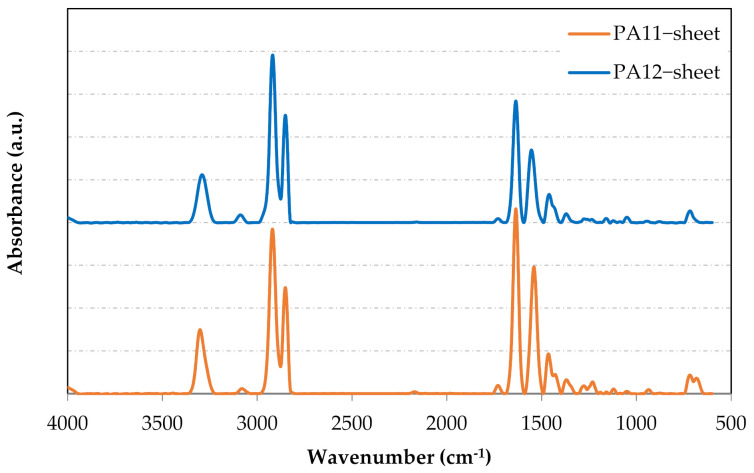
FTIR spectra of hot-pressed PA11 and PA12.

Figure 13 illustrates the obtained spectra for both hot-pressed PAs. All spectra were normalized based on the methyl peak (around 2918 cm^−1^). Very similar peaks were obtained for both PA11 and PA12. The typical bands for PA11 and PA12, i.e., amide I, amide II and amide III, were observed at 1635 cm^−1^, 1540 cm^−1^ and 1275 cm^−1^, respectively. The intense bands for these two polyamides were symmetric and antisymmetric stretching vibrations of C-H at 2850.7 cm^−1^ and 2918.2 cm^−1^, respectively. Furthermore, the characterization bands in the region from 3500 cm^−1^ to 3000 cm^−1^ belong to the hydrogen-bonded N-H stretching (3080 cm^−1^) and OH/NH groups (3400–3100 cm^−1^). A shoulder at 1429 cm^−1^ represents the CH_2_ scissor vibration. Curve-fitting results are listed in Table 5. Both spectra showed a small peak corresponding to the carboxyl group (O-C=O). This peak can be used to provide information about possible acid hydrolysis in the material.

### 3.5. Durability Test

**Figure 14:**
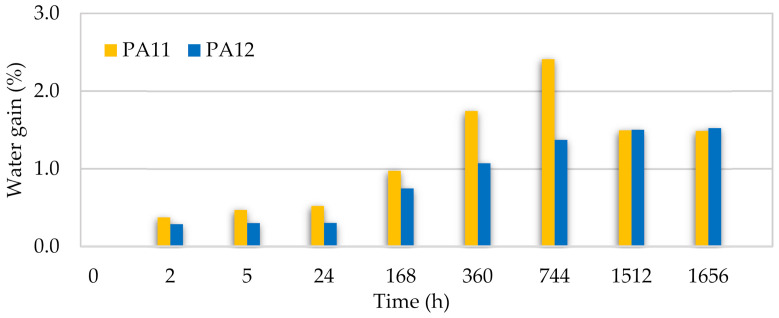
Water gain of PA11 and PA12 at different aging times.

**Figure 15:**
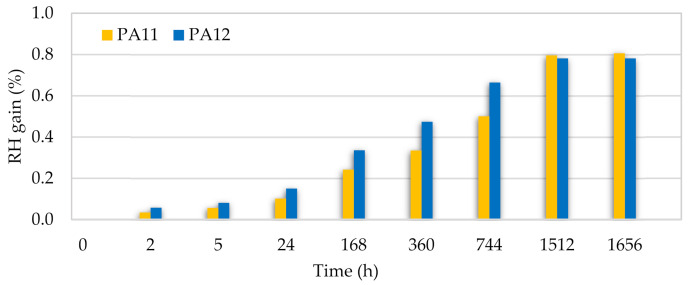
Moisture gain of PA11 and PA12 at different aging times.

Weight gain measurements after the durability test for each aging time are shown in Figure 14 and Figure 15. For both PAs, there is an increase in the water/moisture gain% when increasing the aging time. When approaching the saturation level, because the diffusion flux became zero, less water was absorbed until the water and moisture gain remained constant. After around two months, both PAs had the same saturation level for water and moisture absorption, i.e., 1.5% and 0.8%, respectively. These values show a resistance improvement compared to other reported manufacturing techniques such as laser sintering [13,90] and injection molding [91], which can be explained by the low porosity of the polymers processed by the hot press.

However, consistent with previous reports [58,92], PA12 absorbs the least water among all polyamides at each aging time due to the lower concentration of amides in the polymer chain. Moreover, the overall hydrophobicity of the polyamide increases with a higher repeating unit length, which is equivalent to an increase in the methylene:amide group ratio per repeat unit. The higher degree of crystallinity in PA12 may have been another underlying cause for the lower water absorption since water is absorbed in the amorphous regions, where the hydrogen bonds are free to interact with moisture [60].

In order to investigate the effect of absorbed water and moisture on the absorbance bands of PAs, FTIR-ATR analysis was performed on the test specimens up to the saturation level. Since the water absorbance bands are overlapped with amine bands (3400–3100 cm^−1^), it is impossible to confirm whether there was an amine reaction or free water absorption from the gathered FTIR spectra in relation to this band. However, based on the peaks’ intensity in the regions of 3400–3100 cm^−1^ (OH and NH stretching) and 1750–1500 cm^−1^ (amide/amine N-H deformation, C=N stretching and C-N stretching of amides), it is possible to track the water absorption effects. The water molecules are first bound to the polymer by forming hydrogen bonds with the C=O and the −NH. Then, the remaining water diffuses into the free volume, making it impossible to detect free absorbed water in the FTIR spectra.

**Figure 16:**
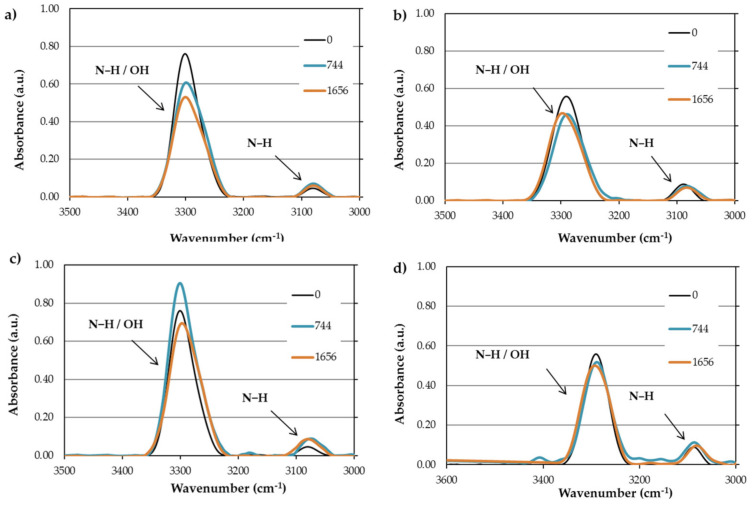
Comparison of OH/NH band between PA11 and PA12 after water and moisture absorption: (**a**) PA11-W; (**b**) PA12-W; (**c**) PA11-RH; (**d**) PA12-RH.

**Figure 17:**
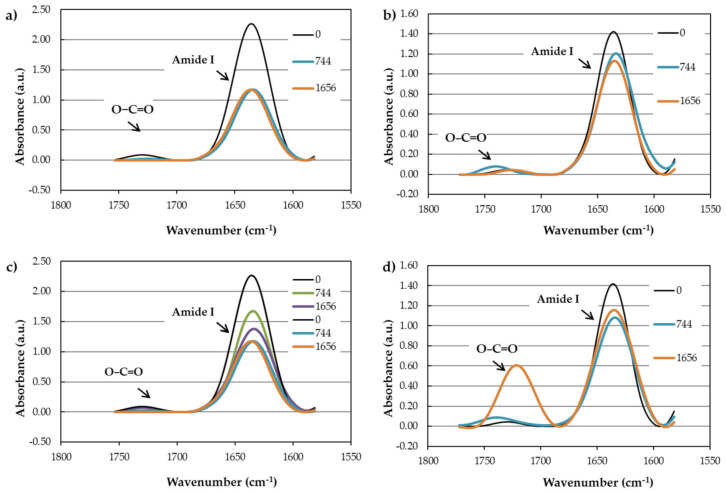
Comparison of amide I band between PA11 and PA12 after water and moisture absorption: (**a**) PA11-W; (**b**) PA12-W; (**c**) PA11-RH; (**d**) PA12-RH.

Figure 16 and Figure 17 compare the mentioned peaks for PA11 and PA12 after the durability test for 774 h and saturation level. To better interpret the peaks, the area under each curve was measured (Tables S3–S6 and Figures S1–S4 in the Supplementary Material). In Figure 16a–d, the peaks assigned to OH/NH stretching (PA11-W/RH: 3306 cm^−1^ and PA12-W/RH: 3292 cm^−1^) decreased upon the durability test compared to the neat PAs. The only exception is that the absorbing band belonging to 774 h in PA11-RH had a 39% increase in curve area, which might be due to the reversible reaction of water with amide groups.

In addition, Figure 17 reveals that the peaks associated with amide I (C=O bonds at 1635 cm^−1^) decreased after absorbing water and moisture. This reduction for PA11 was higher in both cases of water (49%) and moisture (39%) absorption compared to PA12 (13% and 19% for water and moisture, respectively), meaning a higher tendency of the amide group in PA11 to react with absorbed water and form a hydrogen bond. This implies a decrease in molecular weight and accordingly a destructive hydrolysis process, because hydrolysis leads to chain scissions, contributing to lowering the molecular weight [93]. In other words, a decrease in molecular weight encourages a plasticizing effect, which interrupts the intermolecular hydrogen bonding and increases segmental motion [9]. Although the plasticizing effect is more evident at high temperatures [94], it also occurs at low temperatures with embrittlement effects in PAs [95].

**Figure 18:**
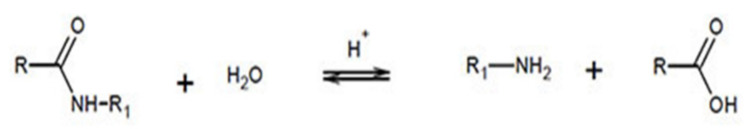
Reversible reaction of an amide in an acid medium in polyamides.

Since the deionized water used in the durability test had a pH value of around 6, the acid hydrolysis process was expected [38,96]. According to Figure 18, after the reaction of water with amide groups, hydrolysis leads to the destruction of amides by chain scission [97]. Each chain scission forms an amine and carboxylic acid end-group as reaction products. Amides hydrolyze slowly due to their basic leaving groups as well as the weak nucleophilicity of water. The determining step of the reaction rate in this process is the formation of the tetrahedral intermediate by protonation of carbonyl oxygen and subsequently the nucleophilic attack of the water. This step is followed by the transfer of a proton from water to the amine. Then, the tetrahedral intermediate breaks down into carboxylic and amine groups. This process is a reversible reaction, meaning that the amine end can attack the carboxylic end and create a longer-chain polymer [59,98].

The amide hydrolysis process can be verified in the FTIR spectra; on the one hand, the O−C=O peak (1716 cm^−1^) intensity increased (Figure 17), especially in PA12, after water immersion and moisture absorption. On the other hand, bonded NH at 3082 cm^−1^ increased in all tested PAs.

All the PAs that were subjected to water immersion and relative humidity were evaluated by DSC analysis. The thermal properties after each aging time were measured and are shown in Table 6. Different parameters led to almost unchanged thermal properties. The distribution of values was evaluated by analysis of variance (ANOVA). The *p*-value of ANOVA was lower than the confidence interval (95% or alpha = 0.05), which proves similarity of all data. Firstly, the water/moisture gain was less than 1.5%, which is a relatively low amount; consequently, the hydrolysis process would be slight. Secondly, the durability test was performed at room temperature; thus, there was no phase transformation within the structure of the polyamides. In this regard, the only visible change was the slight decrease in T_g_ during the aging, which was assumed to result from the hydrolysis, which promotes the mobility of amorphous chains. The differences found in crystallinity show that the process is reversible; when the crystallinity decreases, the amorphous phase increases and vice versa. Although the differences are minimal, this may be due to measurement error since this analysis cannot be repeated more than three times to avoid changes in the sample.

The results of the Shore-D hardness measurements are presented in Table 7. As can be seen, the hardness values are nearly the same before and after aging for both PA11 and PA12, meaning that water and moisture did not affect the hardness of specimens. It can be concluded that the water/moisture absorption was not sufficient to deteriorate the hardness of the polymers. This means that there was no plasticizing effect. It shows that acid hydrolysis was slight; consequently, the mechanical properties were not compromised. Since abrasive resistance is an essential property for tribological applications, the Shore-D test confirmed the hardness stability of PA11 and PA12 in applications that require tribological properties in humid or wet environments.

## 4. Conclusions

In this study, hot-pressed PA11 and PA12 sheets were characterized employing XRD, DSC, FTIR and SEM imaging. Furthermore, the manufactured PAs were compared in terms of tensile properties, hardness and durability. Compared to the other manufacturing techniques (e.g., SLS and injection molding), the hot press method still has some benefits, such as high molding pressure, which can lead to superior material properties. The XRD and DSC results revealed that the predominate crystal phases in PA11 and PA12 are the triclinic α-phase and pseudohexagonal γ-phase, respectively, resulting in 21% and 49% crystallinity. By comparing the stress–strain curves and SEM micrographs, it could be concluded that PA12 has superior tensile properties with ductile behavior, which resulted in a waviness fracture surface consisting of folds or rounded kinks. Due to the presence of more crystalline phases, which provided more ductility, the tensile properties of PA12 were significantly improved with respect to previous works. On the contrary, PA11 with quasi-brittle behavior had a smooth fracture surface consisting of tilted and twisted lamella.

After two months of aging, both PA11 and PA12 had relatively low water (1.5 wt%) and moisture (0.8 wt%) gain under water and 50% relative humidity conditions. A much greater reduction in the C=O peak intensity in the FTIR spectra of PA11 after the durability test proved the greater tendency of this polyamide with respect to PA12 in terms of water/moisture absorption. Furthermore, a decrease in molecular weight and increase in bonded −NH implied slight hydrolysis. However, a low amount of absorbed water/moisture and hydrolysis did not deteriorate the dimensional stability or mechanical properties of the polyamides, such as hardness. In brief, although the bio-based PA11 is preferable over oil-based PA12 from an environmental perspective, PA12 is a more exploitable option due to the better mechanical performance and durability. The aim of future work is to employ these two commercial polyamides as matrices of hybrid composites to achieve high-performance materials.

## Figures and Tables

**Table 1:** Tensile properties of PA11 and PA12.

	E (MPa)	Ultimate Strength (MPa)	Elongation at Break (%)
PA11	1762.2 ± 138.3	20.0 ± 3.8	4
PA12	2964.3 ± 102.8	64.0 ± 3.2	200

**Table 2:** Comparison of tensile properties between the present study and previous works.

	Manufacturing Technique	E (GPa)	Ultimate Strength (MPa)	Elongation at Break (%)	χ_c_ (%)	Ref.
PA11	Hot press	1.7	20	4	23	Present work
	Hot press	1.0	44.9	26	27	[68]
	Hot press	0.3	32	252	-	[69]
	SLS	1.3	49.6	-	28	[7]
	Injection molding	-	39	Without break	-	[70]
	Extrusion	1.2	36.5	235	-	[71]
	Injection molding	1.3	48	137	-	[72]
PA12	Hot press	2.9	64	200	49	Present work
	Hot press	1.7	51.2	163		[73]
	Hot press	0.8	39.6	24	-	[74]
	HP jet fusion	1.2	47	19	26	[75]
	Injection molding	1.3	35	230	-	[76]
	SLS	1.7	42.2	7	-	[73]
	SLS	1.4	33	-	-	[7]

**Table 3:** XRD experimental peak positions in PA11 and PA12.

	Phase	2θ°
PA11	α (triclinic)	20.5
		23.7
PA12	γ (hexagonal)	21.45

**Table 4:** Thermal properties of studied polyamides by DSC.

PA11				PA12			
	**1st Heating**	**Cooling**	**2nd Heating**		**1st Heating**	**Cooling**	**2nd Heating**
T_g_ (°C)	44.49		45.95	T_g_ (°C)	42.77		49.29
T_m_ (°C)	193.90		188.50	T_m_ (°C)	178.86		178.05
T_c_ (°C)		162.52		T_c_ (°C)		144.07	
∆H (J/g)	51.87	39.27	47.86	∆H (J/g)	46.52	57.15	47.15
χ_c_ (%)	23.16	17.53	21.37	χ_c_ (%)	49.48	60.16	49.63

**Table 5:** Curve-fitting results of FTIR spectra for PA11 and PA12 [89].

Frequency (cm^−1^)	Vibration
3301	N−H stretching strong band/OH
3082	NH groups weak band
2918	CH_2_ asymmetric stretching
2850	CH_2_ symmetric stretching
1732	O−C=O
1635	Amide I, C=O stretching
1550	Amide II, C- stretching + C=O in-plane bending
1465	CH_2_ bending asym
1367	CH_2_ bending sym
1275	Amide III, NH−O stretching
1226	C−O−C streching sym/CH_2_ bending
1111	CH_3_ rocking
934	C−C(O) stretching mode (amide IV)
721	CH_2_ rocking/C=O deformation
678	NH out-of-plane mode (amide V)

**Table 6:** Thermal properties of studied polyamides by DSC after the durability test.

Property	Sample	0 h	2 h	24 h	360 h	744 h	1656 h
T_g_ (°C)	PA11-HR	44.49	42.34	40.62	39.34	39.78	39.89
T_m_ (°C)		193.9	194.98	197.89	194.3	193.28	195.16
∆H (J/g)		51.87	52.55	45.7	51	48.22	57.83
χ_c_ (%)		23.16	23.46	20.4	22.77	21.53	25.82
T_g_ (°C)	PA11-WA	44.49	42.57	42.83	42.07	40.59	41.22
T_m_ (°C)		193.9	195.02	194.67	194.62	195.25	195.16
∆H (J/g)		51.87	50.27	44.88	44.84	48.43	57
χ_c_ (%)		23.16	22.44	20.03	20.02	21.62	25.45
T_g_ (°C)	PA12-HR	42.77	41.89	41.61	39.84	37.71	39.33
T_m_ (°C)		178.86	180.94	185.94	184.14	182.96	184.88
∆H (J/g)		46.52	50.13	40.11	33.26	47.09	45.83
χ_c_ (%)		49.48	52.77	42.22	35.01	49.57	48.25
T_g_ (°C)	PA12-WA	42.77	43.35	47.03	48.01	40.92	39.95
T_m_ (°C)		178.86	184.03	182.66	182.06	182.46	184.2
∆H (J/g)		46.52	43	34.56	46.17	39.49	46.64
χ_c_ (%)		49.48	45.26	36.38	48.6	41.57	49.09

**Table 7:** Shore-D hardness values (±2) for PA11 and PA12 at different aging times.

Time (h)	PA11-RH	PA12-RH	PA11-W	PA12-W
0	74	73	74	73
24	74	71	74	73
360	74	72	74	73
744	74	72	74	71
1656	73	74	72	72

## Supplementary Materials

The following are available online at https://www.mdpi.com/article/10.3390/polym13203553/s1, Table S1: Thermal properties of PA11 powder analyzed by DSC; Table S2: Thermal properties of PA12 powder analyzed by DSC; Figure S1: Comparison of OH/NH and amide I bands for PA11-W at different aging times; Table S3: Calculated area assigned to each FTIR peak for PA11-W; Figure S2: Comparison of OH/NH and amide I bands for PA11-RH at different aging times; Table S4: Calculated area assigned to each FTIR peak for PA11-RH; Figure S3: Comparison of OH/NH and amide I bands for PA12-W at different aging times; Table S5: Calculated area assigned to each FTIR peak for PA12-W; Figure S4: Comparison of OH/NH and amide I bands for PA12-RH at different aging times; Table S6: Calculated area assigned to each FTIR peak for PA12-RH.
