# Supplementary material for: Comparative Characterization of Hot-Pressed Polyamide 11 and 12: Mechanical, Thermal and Durability Properties
Source: Polymers (Basel). 2021 Oct 15;13(20):3553. doi: 10.3390/polym13203553 (PMC8537549; doi:10.3390/polym13203553)
Supplement: Supplementary file 1 [file polymers-13-03553-s001.zip › polymers-1416926-supplementary.pdf]

Supporting information

Comparative characterization of hot-pressed polyamide 11 and 12: mechanical, thermal and durability properties

M. Bahrami[1], J. Abenojar[1,2], M.A. Martínez[1]
[1]Materials Science and Engineering Department, University Carlos III de Madrid, Leganes, Spain
[2]Mechanical Engineering Department, ICAI, Universidad Pontificia Comillas, Madrid, Spain
* Corresponding author: mbahrami@ing.uc3m.es

Table S1: Thermal properties of PA11 powder analyzed by DSC

	1st Heating	Cooling	2nd Heating
**T_g (°C)**	53.97	-	55.03
**$\Delta H_{relax.}$(J/g)**	1.21	-	-
**T_w (°C)**	91.65	-	-
**T_m (°C)**	192.25	-	191
**T_c (°C)**	-	158.95	-
**ΔH (J/g)**	44.86	39.57	51.68
**χ_c (%)**	20.03	17.66	23.07

Table S2: Thermal properties of PA12 powder analyzed by DSC

	1st Heating	Cooling	2nd Heating
**T_g (°C)**	55.32	-	56.99
**DH_{water} (J/g)**	1.49	-	-
**T_w (°C)**	83.36	-	-
**T_m (°C)**	184.26	-	182.75
**DH (J/g)**	47.69	-	55.59
**T_c (°C)**	158.61*	140.46	-
**DHc (J/g)**	1.74*	53.79	-
**χ_c (%)**	50.2	56.62	58.51

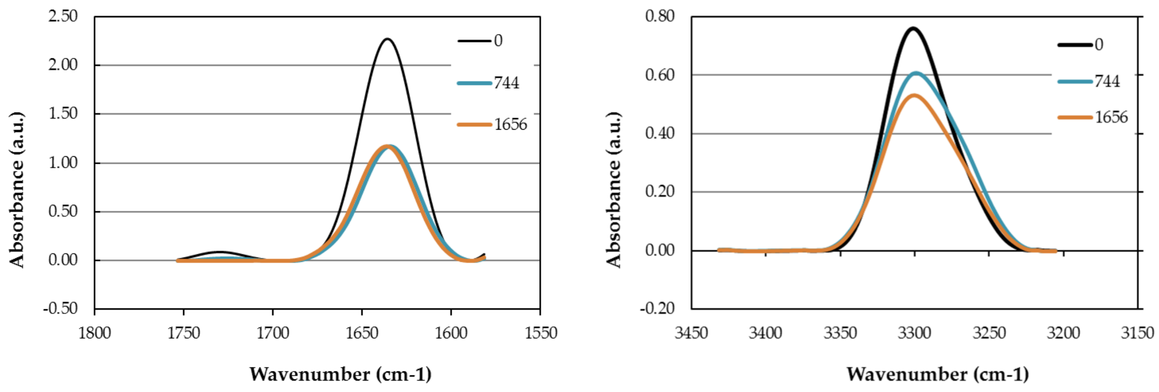

Figure S1: Comparison of OH/NH and amide I bands for PA11-W at different aging times

Table S3: Calculated area assigned to each FTIR peaks for PA11-W

Time (h)	Area	Peak	%
0	46.983032	3306	-
	109.406387	1635	-
744	40.4783678	3304	-14
	56.3515581	1635	-48
1656	35.4565545	3304	-25
	55.7435171	1635	-49

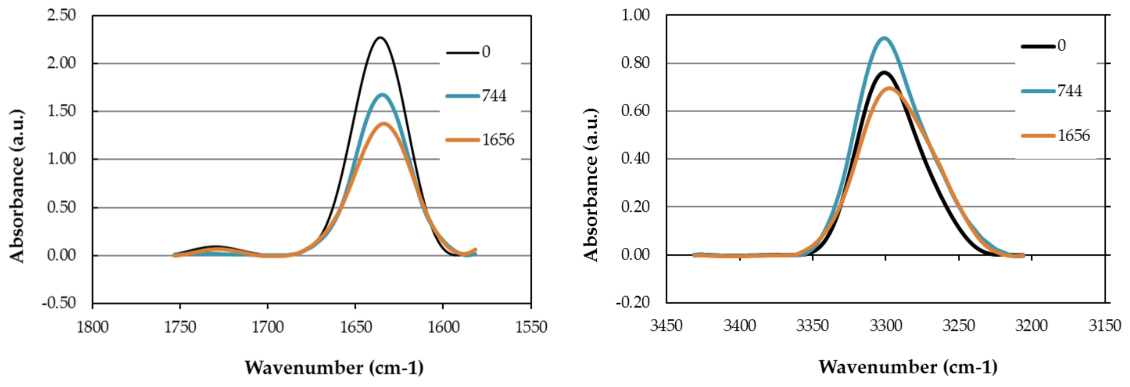

Figure S2: Comparison of OH/NH and amide I bands for PA11-RH at different aging times

Table S4: Calculated area assigned to each FTIR peaks for PA11-RH

Time (h)	Area	Peak	%
0	46.983032	3306	-
	109.406387	1635	-
744	65.3443497	3074	39
	80.7633659	1635	-26
1656	47.3723562	3082	1

	66.3917079	1635	-39

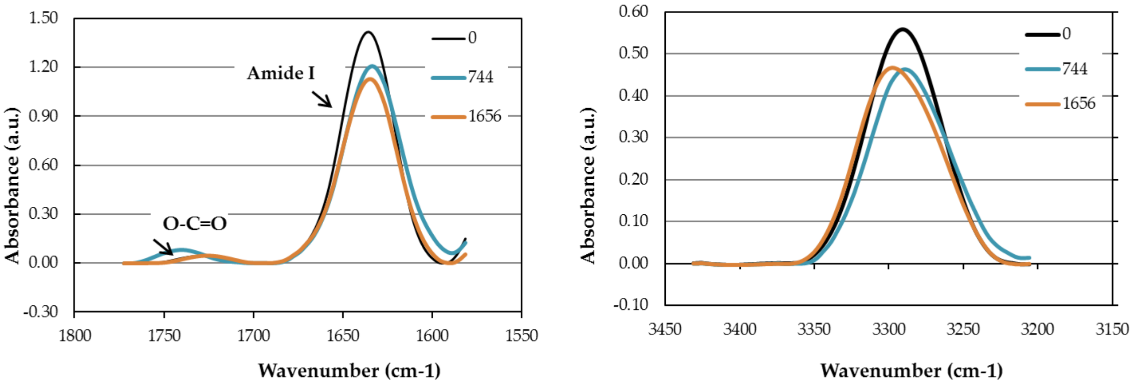

Figure S3: Comparison of OH/NH and amide I bands for PA12-W at different aging times

Table S5:Calculated area assigned to each FTIR peaks for PA12-W

Time (h)	Area	Peak	%
0	37.8556171	3292	-
	59.957971	1639	-
	0.81490237	1732	-
744	32.3649299	3292	-15
	56.6839088	1638	-5
	1.7148249	1739	110
1656	31.9312965	3302	-16
	52.1830946	1633	-13
	0.91806917	1724	13

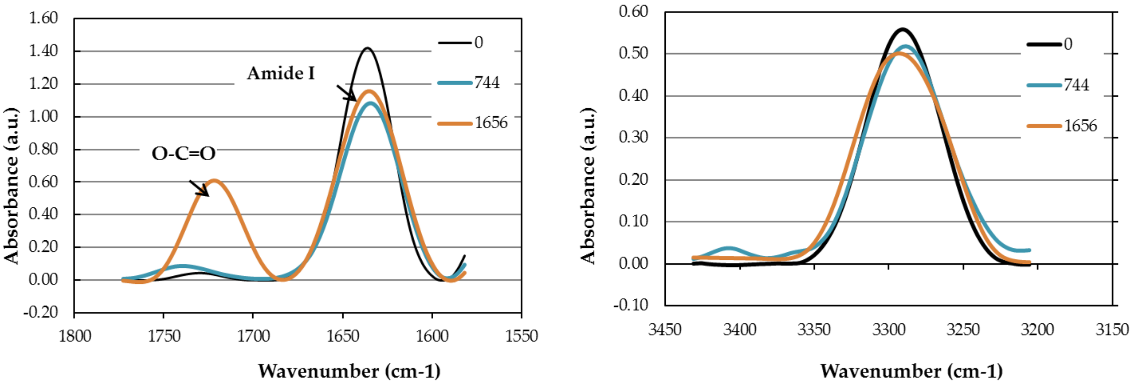

Figure S4: Comparison of OH/NH and amide I bands for PA12-RH at different aging times

Table S6: Calculated area assigned to each FTIR peaks for PA12-RH

Time (h)	Area	Peak	%
0	37.8556171	3292	-
	59.957971	1639	-
	0.81490237	1732	-
744	38.6684452	3298	2
	52.0158558	1635	-13
	3.03641398	1738	273
1656	38.6684452	3298	2
	48.6039726	1635	-19
	20.5115793	1722	2417
